# On the importance of intersectionality: understanding how experienced, anticipated and internalized stigma intersect in people with non-normative identities

**DOI:** 10.3389/fsoc.2026.1664450

**Published:** 2026-03-03

**Authors:** Elise Wuyts, Kaat Schellen, Minne Van Den Noortgate, Violette Coppens, Wim Huys, Alana Schuerwegen, Samuel Coenen, Kris Goethals, Manuel Morrens

**Affiliations:** 1 Faculty of Medicine and Health Sciences, Collaborative Antwerp Psychiatric Research Institute, University of Antwerp, Antwerp, Belgium; 2 University Department of Psychiatry, Duffel, Belgium; 3 University Forensic Centre, Antwerp University Hospital, Edegem, Belgium; 4 Centre for General Practice, Faculty of Medicine and Health Sciences, University of Antwerp, Antwerp, Belgium

**Keywords:** BDSM, consensual non-monogamy, intersectionality, LGBTQIA+, stigma, awareness

## Abstract

**Introduction:**

Recent years have seen a growing body of work on sexual and gender minority groups, as well as a rising interest in the diversity of relationship structures, both sexual and romantic. The increased visibility and associated counter-movement also impact the stigmatization to which these minority groups are subjected. This is in turn correlated with increased minority stress which has been linked to numerous mental, physical and economic problems. The overlap found between these different minority groups underlines the importance of understanding the complex interplay of stigmatizing factors through the lens of intersectionality.

**Methods:**

A cross-sectional survey was conducted in Belgium in the Dutch language to compare four non-normative groups (sexual orientation, gender identity, sexual relationships and romantic relationships) with each other. Three different scales were used, yielding four stigma scores: (1) the experienced stigma scale, (2) the anticipated stigma scale with two subscores of (2a) anticipated stigma in daily life and (2b) anticipated stigma in relationships, and (3) internalized stigma scale.

**Results:**

A total of 2,576 were included in analyses, of which 519 individuals (20%) identified as non-normative, with 425 belonging to only one category. All of the minority groups included in this study experienced stigmatization because of their non-normative identity, though differences were found in the type of stigma they experienced and the extent to which they experienced stigma compared to the other non-normative groups. Having multiple non-normative identities resulted only in increased anticipated stigma.

**Discussion:**

The results found in this study are comparative to other research. Differences in stigmatization may be explained by differences in visibility and stigma symbols, with some identities being more concealable than others, and differences in societal acceptance in Belgium. The current political landscape underlines the importance of this research, which addresses the need to examine combinations and the interactions between different types of stigma and different non-normative identities.

## Introduction

Recent years have seen a growing body of work on sexual and gender minority groups, such as those belonging to the LGBTQIA+ (lesbian, gay, bisexual, transgender, queer/questioning, intersex, asexual/aromantic and others) community. This umbrella term has evolved over time as a way to foster inclusivity and recognize the spectrum of sexual orientation and gender identity (Kuney et al., 2025). Sexual orientation broadly refers to patterns of emotional and sexual attraction, encompassing not just who individuals are drawn to, but also the behaviors and relationships that stem from those attractions (Ventriglio and Bhugra, 2019). Gender identity, meanwhile, involves an individual’s internal sense of self and how this aligns—or does not align—with societal expectations of masculinity and femininity, as expressed through appearance, behavior, and interests (Winter et al., 2016).

Alongside expanding understandings of identity, there has also been growing interest in the diversity of relationship structures, both sexual and romantic. Sexual relationships are typically characterized by experiences of physical intimacy and erotic connection. However, practices such as open relationships or BDSM (bondage and discipline, dominance and submission, sadism and masochism) challenge conventional assumptions of sexual behavior. Although BDSM is not exclusively a sexual experience, it is often grouped within this domain and refers to a physical, psychological and sexual role-play involving the exchange of power between consensual participants (De Neef et al., 2019). Romantic relationships are also often framed within a normative model—particularly monogamy—which remains the dominant reference point in many Western societies (Lecuona et al., 2021). Yet many individuals experience or pursue alternatives, such as consensual non-monogamy (CNM) or polyamory, which involve maintaining multiple romantic and/or sexual partnerships with the informed consent of all involved (Rubel and Burleigh, 2020). In practice, alternative sexual and romantic relationships are often discussed together, as many romantic relationships have a sexual component and vice versa (Traeen and Thuen, 2022). In this study we have opted for further dividing romantic and sexual relationships into two separate groups to account for the diversity of the spectrum of relationship structures.

Interestingly, significant overlap can be found between these different minority groups. For instance, several studies suggest that LGBTQIA+ people are more likely to be involved in BDSM relationships (Sprott, 2023) and to have experience with CNM relationships (Traeen and Thuen, 2022), BDSM in turn was found to be associated with both non-heterosexual and non-monogamous relationships (Brown et al., 2020). Taken together, these evolving frameworks reflect a growing recognition that individual identities and relational lives are not easily confined to binary categories or conventional scripts. Instead, they unfold across a spectrum shaped by personal identity, cultural norms, and shifting social values.

This increase in academic and public discourse also impacts the stigmatization to which these minority groups are subjected. Sexual and gender minorities have been historically severely stigmatized (Drabish and Theeke, 2022; Henderson et al., 2022), as have those individuals exploring alternative sexual or romantic relationships (Moors et al., 2021; Schuerwegen et al., 2020). Stigma can be described as “the situation of the individual who is disqualified from full social acceptance” (Goffman, 1963). It is a negative social marker that has a profound impact on the way people see themselves and are seen by others and it can lead to significant stress in minority groups.

According to the minority stress theory by Meyer (2003), three processes must be considered in which stigma leads to stress: (1) experiencing stressful events and conditions (i.e., experienced stigma), (2) anticipation of such events and the vigilance this requires (i.e., anticipated stigma), and (3) internalizing these negative societal attitudes (i.e., internalized stigma). This last process of internalization may be defined as a subjective process, embedded within a socio-cultural context, which may be characterized by negative feelings (about self), maladaptive behavior, identity transformation or stereotype endorsement” (Livingston and Boyd, 2010). Quinn et al. (2015) point out that the relationship between experienced stigma and internalized stigma is also mediated by anticipated stigma. The kind of stigma that people are subjected to also depends on the way in which their identity is concealable. This is done by for instance attempting to hide symbols associated with their stigmatized identity. Stigma symbols may refer to the signs in which social information about a stigmatized identity is conveyed (Goffman, 1963). For instance, people may choose to hide their sexual orientation or BDSM preference in certain settings, but many TGNC people cannot hide that they are not cisgender and may therefore suffer more from experienced stigma.

Minority stress has a significant impact on both physical health (Flentje et al., 2020) and mental health (Mezza et al., 2024), with stigma specifically being linked to substance abuse, depression, anxiety disorders and post-traumatic stress disorders (PTSD) (Gamariel et al., 2020; Jefferson et al., 2013; Velasco, 2022). Additionally, stigma leads to increased economic burden (Furuya, 2002) and reduced access in terms of housing, work, education, and qualitative healthcare (Henderson et al., 2022; Velasco, 2022). Research on this topic becomes even more relevant considering that interventions implemented to decrease stigma are often not based on empirical evidence (Bos et al., 2013).

The previously mentioned overlap between the different minority groups underlines the importance of understanding the complex interplay of stigmatizing factors through the lens of intersectionality, which focuses on how multiple systems of oppression interact with each other, such as sexism and racism (Harari and Lee, 2021). This not only provides context for these overlapping identities, but also connects it to the societal structures of privilege and oppression (Buchanan and Wiklund, 2021). This is especially relevant in the current political climate where more conservative and anti-LGBTQIA+ legislation is on the rise, both on an international scale and in Belgium specifically (Mezza et al., 2024; Verlooy, 2024).

For this reason, the current study will investigate stigma among the four different yet overlapping minority groups, namely those who identify as non-normative in regards to sexual orientation, gender identity, sexual relationships and romantic relationships. We have chosen to investigate not only experienced stigma, but also anticipated and internalized stigma in accordance with the minority stress theory of Meyer (2003) to explore these relationships more fully. We hypothesize that stigmatization will present differently between the non-normative groups depending on expected external visibility and stigma symbols, as well as social acceptance within Western society. We further predict higher anticipated and internalized stigma if an individual also scores higher on the experienced stigma scale. Finally, we expect that individuals who identify with multiple non-normative identities will experience higher levels of stigma.

## Methods

### Study design

In March 2023, a cross-sectional survey was conducted online through Bilendi, a market research and polling agency with a database of 150,000 Dutch-speaking Belgian citizens as a representative sample of the general population. People were asked to participate in the survey through email with the aim of including 2,000 participants in four predetermined age groups (18–25; 26–39; 40–59; 60 and above). The invitation contained information on the time needed to complete the survey (12 min) and the incentive for participation (110 points for the Bilendi web shop, equivalent to €0.7 per survey). Information on the content of the survey was presented only after opening the link, together with an informed consent form. This study is approved by the ethical committee of the University of Antwerp in accordance with the ethical standard of the 1964 Helsinki Declaration and later amendments.

### Survey

The survey, written in Dutch, included a series of questions to gauge demographic factors (age, gender identity, sexual identity, education, living environment). The participants were then asked whether they considered themselves to be non-normative in regards to gender identity, sexual orientation, sexual relationships and romantic relationships with a short explanation for each option. They also had the possibility of checking a box “other” which they could fill out themselves, or a box indicating they considered themselves normative on all four aspects mentioned above. Multiple boxes could be checked. Only participants who had checked at least one non-normative or “other” box, were presented with the three stigma questionnaires.

These non-normative participants completed three different stigma scales, yielding four stigma scores: (1) The experienced stigma scale (ESS) is based on the Everyday Discrimination Scale (Williams et al., 1997) and contains 9 items with a chronicity-based 6-likert scale response category ranging from never to almost every day. Participants were asked how often they experienced any of the 9 situations because of their non-normative identity (e.g., “I am threatened or harassed”). (2) The anticipated stigma scale (ASS) is based on the 15-item scale used by Quinn and Chaudoir (2009) with an internal reliability of 0.95 and consisting of two subscales: (2a) 9 items based on the “day-to-day” discrimination scale of Kessler et al. (1999) and (2b) 6 further items focusing on relational concerns. All items were scored on a 5-likert scale response category ranging from highly unlikely to highly likely. Participants were asked how probable they would find each situation if their non-normative identity would be known (e.g., “I would be treated with less respect than other people”; “friends would ignore or avoid me”). (3) The internalized stigma scale (ISS) is based on the 4-item scale used by Quinn et al. (2015) and scored on a 5-likert scale response category ranging from disagree completely to agree completely. Participants were asked to what extent they agreed with the four statements gauging self-stigmatization (e.g., “I feel guilty about my non-normative identity”).

### Statistical analyses

Statistical analyses were conducted using SPSS version 29.0. The between-group comparisons for ordinal and nominal data were performed by use of Pearson’s chi square analyses, whereas continuous data comparisons were done by ANOVA analyses. The stigma scales were tested for internal consistency by means of the Cronbach’s Alpha test, which yielded the following results: (1) the ESS had a Cronbach Alpha’s score of 0.950; (2) the ASS had a Cronbach’s Alpha score of 0.955; (3) the ISS had a Cronbach’s Alpha score of 0.937. These scores indicate an excellent internal consistency.

Only those within the non-normative group who identified with a single non-normative identity were included in comparisons between non-normative identities, to avoid interference from the overlapping identities. In accordance with previous research by Michaels et al. (2019), an average score on each stigma scale and subscale was computed and used in further analyses. Because the stigma scales were moderately correlated, we treated them as a multivariate outcome and, in line with current recommendations, used descriptive discriminant analysis (DDA) as the only post-hoc procedure whenever a MANOVA was statistically significant, rather than running multiple univariate ANOVAs (Barton et al., 2016). For each significant effect, DDA extracted a single canonical discriminant function (for dichotomous groupings) summarizing the pattern of group differences across stigma dimensions; we interpreted these functions using Wilks’ lambda and the canonical correlation as overall multivariate effect indices, and used structure coefficients (pooled correlations between each stigma scale and the discriminant function) together with group centroids (mean discriminant scores per group) to determine which types of stigma contributed most to the multivariate separation and in which direction. Assumptions were evaluated by inspecting log determinants of the group covariance matrices, as Box’s *M* test is known to be overly sensitive in large samples (Barton et al., 2016). Each non-normative identity was used as an independent variable in turn and compared to the other identities collectively to explore differences in average stigma scores on the different stigma scales between identities. Lastly, those who identified with a single non-normative identity within the non-normative group were compared to those with multiple identities to explore differences in average stigma scores on the different stigma scales.

## Results

### Distribution of non-normative identities

A total of 2,576 respondents were included in this study. Excluded from analysis were 198 participants with either missing data (*n* = 84) or those with ambiguous answers in regards to sexual orientation (*n* = 114), which involved 67 persons in the non-normative sexual orientation group who identified as heterosexual, 27 persons in the normative sexual orientation group who identified as bisexual and 20 who identified as homosexual.

This left a total of 2,057 respondents who identified as normative and 519 respondents (20% of the 2,576 included participants) who identified with one or more non-normative identities. Of this last group, 208 (40.1%) cited a non-normative gender identity, 166 (32.0%) reported a non-normative sexual orientation, 172 (33.1%) had experience with a non-normative sexual relationship and 92 (17.7%) with a non-normative romantic relationship. There was a considerable overlap between the four categories, with 425 (81.7%) only belonging to one category, 74 (14.4%) belonging to two, 18 (3.5%) belonging to three, and 2 (0.4%) belonging to all four non-normative categories (see Table 1).

**Table 1:** Demographic distribution of non-normative identities.

	Gender identity			Sexual orientation			Sexual relationships			Romantic relationship		
	Normative (*n* = 2,368)	Non-normative (*n* = 208)	Test	Normative (*n* = 2,410)	Non-normative (*n* = 166)	Test	Normative (*n* = 2,404)	Non-normative (*n* = 172)	Test	Normative (*n* = 2,483)	Non-normative (*n* = 92)	Test
Age	46.2 (18.2)	40.7 (17.6)	19.6^***,^^a^	46.3 (18.3)	38.3 (15.1)	29.9^***,^^a^	46.3 (18.3)	38.3 (15.1)	34.3^***,^^a^	46.0 (18.3)	39.7 (16.3)	14.4 ^***,^^a^
Gender identity												
Man	1,152 (49%)	116 (55%)		1,187 (49%)	81 (49%)		1,160 (48%)	108 (63%)		1,210 (49%)	58 (63%)	
			61.3^***,^^b^			73.4^***,^^b^			36.7^***,^^b^			8.5^*,^^b^
Woman	1,209 (51%)	85 (41%)		1,217 (51%)	76 (46%)		1,235 (52%)	58 (34%)		1,260 (51%)	33 (36%)	
Non-binary	4 (0%)	8 (4%)		4 (0%)	8 (5%)		8 (0%)	4 (2%)		11 (0%)	1 (1%)	
Other	2 (0%)	0 (0%)		2 (0%)	0 (0%)		1 (0%)	1 (1%)		2 (0%)	0 (0%)	
Sex orientation												
Heterosexual	2089 (88%)	191 (91%)		2,280 (95%)	0 (0%)		2,173 (91%)	108 (63%)		2,222 (90%)	58 (65%)	
			14.7^*,^^b^			2107.3^***,^^b^			133.8^***,^^b^			63.6^***,^^b^
Heteroflexible	114 (5%)	4 (2%)		98 (4%)	20 (12%)		94 (4%)	23 (14%)		102 (4%)	16 (18%)	
Bisexual	42 (3%)	1 (1%)		0 (0%)	43 (26%)		29 (1%)	14 (8%)		40 (2%)	3 (3%)	
Homoflexible	16 (0%)	3 (1%)		6 (0%)	13 (8%)		15 (1%)	3 (2%)		15 (0%)	3 (3%)	
Homosexual	74 (3%)	4 (2%)		0 (0%)	77 (47%)		60 (2%)	17 (10%)		70 (3%)	7 (8%)	
Other	22 (1%)	6 (3%)		17 (1%)	11 (7%)		22 (1%)	6 (3%)		25 (1%)	3 (3%)	
Living space												
City	277 (12%)	35 (17%)		274 (11%)	38 (23%)		283 (12%)	29 (17%)		299 (12%)	13 (14%)	
			5.7^b^			20.1^***,^^b^			5.1^b^			2.9^b^
Suburbs	893 (38%)	81 (39%)		914 (38%)	60 (36%)		906 (38%)	68 (39%)		947 (38%)	27 (29%)	
Rural	1,198 (50%)	92 (44%)		1,222 (51%)	68 (41%)		1,215 (50%)	75 (44%)		1,238 (50%)	52 (57%)	
Education level												
Elementary	298 (13%)	59 (28%)		344 (14%)	13 (8%)		336 (14%)	21 (12%)		355 (14%)	2 (2%)	
			42.5^***,^^b^			7.0^*,^^b^			0.5^b^			11.9^**,^^b^
Secondary	937 (39%)	80 (38%)		954 (40%)	62 (38%)		946 (39%)	71 (41%)		980 (40%)	37 (40%)	
Higher	1,133 (48%)	70 (34%)		1,113 (46%)	90 (54%)		1,122 (47%)	80 (47%)		1,149 (46%)	53 (58%)	

^*^p-value = <0.05; ^**^p-value = <0.01; ^***^p-value = < 0.001.

a Analysis of variance (ANOVA) (*F*-value) for continuous variables.

b Crosstabs Pearson Chi-square (*F*-value) for non-continuous variables.

Compared to normative-identifying individuals, significant age differences were present for all non-normative identities. These differences were in all cases driven by a slightly older age for the normative identifying participants. Compared to the normative identity group, each of the non-normative identities were also associated with a significantly higher distribution of male and non-binary gender identities. As can be expected, the participants with non-normative sexual and romantic relationships had significantly higher non-heterosexual identities, though this difference was less pronounced in those with a non-normative gender identity. Those with a non-normative sexual orientation were more likely to live in an urban environment, though no significant difference was found for other non-normative groups. The education level was significantly lower for those with a non-normative gender identity, while those with a non-normative sexual orientation or romantic relationship had a significantly higher education level compared to their normative counterparts.

### Associations between stigma scales for different non-normative identities

To examine stigma associated with each of the four non-normative identities, analyses were limited to individuals who reported only a single non-normative identity (*n* = 425). Those with multiple non-normative identities and those with only normative identities were excluded from these analyses. DDA’s were conducted to examine group differences across four stigma dimensions: experienced stigma (ESS), the two subscales of anticipated stigma (ASS–Daily Life and ASS–Relationships), and internalized stigma (ISS).

The DDA for non-normative gender identity indicated that the single canonical discriminant function significantly differentiated participants with a non-normative (*n* = 186) versus normative (*n* = 239) gender identity within the non-normative group [Wilks’ *λ* = 0.859, *χ*^2^(4) = 50.09, *p* < 0.001]. The canonical correlation was 0.375, suggesting that approximately 14% of the variance in the multivariate stigma composite was explained by gender identity status. Structure coefficients (ordered by absolute magnitude): ISS: 0.788; ESS: 0.500; ASS-relationships: 0.183; ASS-daily life: 0.096. As such, internalized stigma contributed most strongly to the group separation, followed by experienced stigma. Anticipated stigma contributed minimally. Group centroids (normative = −0.36, non-normative = 0.46) indicated higher scores on this stigma composite among participants with a non-normative gender identity, reflecting particularly elevated internalized and, to a lesser extent, experienced stigma in this group (see Table 2).

**Table 2:** Associations between stigma scales and non-normative identities.

	Gender identity			Sexual orientation			Sexual relationships			Romantic relationships			Number of categories		
	Normative (*n* = 211)^a^	Non-normative (*n* = 161)^a^	Test^b^	Normative (*n* = 264)^a^	Non-normative (*n* = 108)^a^	Test^b^	Normative (*n* = 305)^a^	Non-normative (*n* = 67)^a^	Test^b^	Normative (*n* = 336)^a^	Non-normative (*n* = 36)^a^	Test^b^	One (*n* = 372)^a^	Two or more (*n* = 77)^a^	Test^b^
Experienced stigma	1.90 (1.04) {1.71–2.02}	2.36 (1.32) {2.23–2.58}	0.859^***^	2.26 (1.27) {2.13–2.40}	1.71 (0.84) {1.49–1.91}	0.799^***^	2.12 (1.19) {1.99–2.25}	2.03 (1.18) {1.74–2.29}	0.936^***^	2.09 (1.19) {1.97–2.22}	2.24 (1.20) {1.77–2.52}	0.999	2.10 (1.19) {1.99–2.21}	1.84 (1.05) {1.59–2.09}	0.915^***^
Anticipated stigma daily life	2.59 (0.96) {2.46–2.71}	2.64 (0.92) {2.51–2.80}		2.62 (0.94) {2.51–2.73}	2.59 (0.95) {2.42–2.77}		2.63 (0.92) {2.53–2.74}	2.52 (1.04) {2.29–2.73}		2.60 (0.95) {2.51–2.70}	2.74 (0.87) {2.38–2.98}		2.61 (0.94) {2.52–2.71}	2.73 (0.99) {2.52–2.94}	
Anticipated stigma relationships	2.53 (0.93) {2.40–2.79}	2.63 (0.95) {2.50–2.79}		2.67 (0.94) {2.56–2.78}	2.34 (0.88) {2.17–2.52}		2.54 (0.92) {2.43–2.64}	2.74 (0.99) {2.51–2.96}		2.56 (0.95) {2.46–2.66}	2.69 (0.85) {2.35–2.96}		2.57 (0.94) {2.48–2.67}	2.74 (0.86) {2.54–2.95}	
Internalized stigma	1.96 (1.01) {1.83–2.10}	2.58 (1.06) {2.42–2.73}		2.47 (1.02) {2.34–2.58}	1.65 (0.90) {1.49–1.86}		2.22 (1.02) {2.11–2.34}	2.25 (1.22) {1.99–2.50}		2.22 (1.08) {2.10–2.33}	2.35 (0.91) {2.01–2.69}		2.23 (1.06) {2.12–2.34}	1.78 (1.06) {1.53–2.01}	

^*^p-value = <0.05; ^**^p-value = <0.01; ^***^p-value < 0.001.

a Mean score (standard deviation) {95% confidence interval}.

b Descriptive discriminant analysis (DDA): Wilks’ *λ*.

Secondly, the DDA for non-normative sexual orientation indicated that the single canonical discriminant function significantly differentiated participants with a non-normative (*n* = 108) versus normative (*n* = 316) sexual orientation within the non-normative group [Wilks’ *λ* = 0.799; *χ*^2^(4) = 73.93; *p* < 0.001]. The canonical correlation was 0.448, suggesting that approximately 20% of the variance in the multivariate stigma composite was explained by sexual orientation status. Structure coefficients (ordered by absolute magnitude): ISS: 0.750; ESS: 0.433; ASS-relationships: 0.362; ASS-daily life: 0.043. As such, internalized stigma contributed most strongly to the group separation, followed by experienced stigma and anticipated stigma in relationships. Anticipated stigma in daily life contributed minimally. Group centroids (normative = 0.32, non-normative = −0.78) indicated lower scores on this stigma composite among participants with a non-normative sexual orientation, reflecting particularly lower internalized and, to a lesser extent, experienced stigma and anticipated stigma in relationships in this group (see Table 2).

Thirdly, the DDA for non-normative sexual relationships indicated that the single canonical discriminant function significantly differentiated participants with a non-normative (*n* = 88) versus normative (*n* = 336) sexual orientation within the non-normative group [Wilks’ *λ* = 0.936; *χ*^2^(4) = 21.67; *p* < 0.001]. The canonical correlation was 0.252, suggesting that only 6% of the variance in the multivariate stigma composite was explained by sexual relationship status. Structure coefficients (ordered by absolute magnitude): ASS-daily life: 0.162; ESS: 0.078; ISS: −0.012; ASS-relationships: −0.345. As such, anticipated stigma in daily life contributed most strongly to the group separation, followed by experienced stigma, although it must be stressed these were modest effects. Internalized stigma and anticipated stigma in relationships contributed minimally. Group centroids (normative = 0.12, non-normative = −0.56) indicated lower scores on this stigma composite among participants with a non-normative sexual relationship, reflecting particularly in lower anticipated stigma in daily life and, to a lesser extent, experienced stigma in this group (see Table 2).

Lastly, the DDA for non-normative romantic relationship indicated that the single canonical discriminant function did not significantly differentiated participants with a non-normative (*n* = 42) versus normative (*n* = 382) romantic relationship within the non-normative group [Wilks’ *λ* = 0.999; *χ*^2^(4) = 0.43; *p* = 0.980] (see Table 2).

### Associations between stigma scales for multiple non-normative identities

Finally, those with a single non-normative identity (*n* = 425) were compared to those with multiple non-normative identities (*n* = 94). The DDA indicated that the single canonical discriminant function significantly differentiated participants with a single non-normative identity versus multiple non-normative identities [Wilks’ *λ* = 0.915; *χ*^2^(4) = 35.84; *p* < 0.001]. The canonical correlation was 0.292, suggesting that approximately 9% of the variance in the multivariate stigma composite was explained by multiple non-normative identity status. Structure coefficients (ordered by absolute magnitude): ISS: 0.578; ESS: 0.306; ASS-daily life: −0.095; ASS-relationships: −0.204. As such, internalized stigma contributed most strongly to the group separation, followed by experienced stigma. Anticipated stigma contributed minimally. Group centroids (single = 0.14, multiple = −0.66) indicated lower scores on this stigma composite among participants with multiple non-normative identities, reflecting particularly lower internalized and, to a lesser extent, experienced stigma in this group (see Table 2).

## Discussion

### Main results

Approximately 20% of the total sample identified with at least one non-normative identity. All of the minority groups included in this study experienced stigmatization because of their non-normative identity, though differences exist in the type of stigma they experience and the extent to which they experience stigma compared to the other non-normative groups. Multiple non-normative identity (i.e., belonging to multiple non-normative groups) resulted in increased anticipated stigma, though not in experienced or internalized stigma.

### Stigmatization of non-normative gender identities

This study found that people with non-normative gender identities comprised about 8.1% of all included participants. The group was generally younger in age, more likely to identify male or non-binary, and had a lower educational level compared to those with a normative gender identity. Further analyses conducted within the non-normative group showed higher average scores of all four kinds of stigma compared to those with other non-normative identities, with internalized stigma and experienced stigma scores contributing most strongly.

These results were comparative to other studies, which found higher rates of stigmatization among transgender and gender non-conforming (TGNC) people: they experienced higher rates of discrimination (Drabish and Theeke, 2022), negative attitudes (Norton and Herek, 2013), mistreatment in healthcare settings (Clark et al., 2025) and violence (Newcomb et al., 2020). Often, TGNC people are more visible in their minority status because of their gender-nonconformity, which may translate to visible markers such as clothing style, hair style, etc. This also puts them at a higher risk for experienced discrimination (Miller and Grollman, 2015) and explains why anticipated stigma is less strongly correlated, seeing as this type of stigma mostly affects those with a concealable stigma identity (CSI).

As Winter et al. (2016) explored, TGNC people often exist at the margins of society, facing violence, exclusion, discrimination and poorer access to health. This may also be observed in this study population with people with non-normative gender identities having a lower education level on average compared to the general population. There are many different hypotheses which may explain these results, among which the link between stigmatization and restricted access to education previously mentioned (Henderson et al., 2022). Recent research has also found a higher drop-out rate for TGNC people in higher education setting, due to factors such as mental health challenges, financial problems and harassment on campus (Liss et al., 2024). As in many parts of the world, Belgium is also seeing a rise in anti-gender and anti-trans mobilizations (Verlooy, 2024) despite its generally pro-LGBTQIA+ legislature. This is important to note, because anti-trans legislation is associated with higher rates of experienced discrimination (Tebbe et al., 2022).

Not only experienced stigma, but also higher rates of internalized stigma were found among TGNC people (Valentine and Shipherd, 2018). This is in turn associated with mental health problems such as substance abuse, eating disorders and increased risk of suicidality (Drabish and Theeke, 2022). Approximately 40% of transgender Americans reported attempting suicide at least once in their lifetime (James et al., 2016). An interesting study from Doyle et al. (2021) researched identity-related resilience factors associated with wellbeing among TGNC people. They found that experiences of discrimination were associated with lower well-being overall, but having a stronger transgender identity moderated this association. Gender identity affirmation was linked to well-being through reinforcing a strong, internalized sense of clarity about individual self-concept, pointing out the need for supportive, identity-affirming social environments.

### Stigmatization of non-normative sexual orientations

The current study found that people with non-normative sexual orientations comprised about 6.4% of all included participants. The group was generally younger in age, more likely to live in an urban environment and had a higher educational level compared to those with a normative sexual orientation. Further analyses conducted within the non-normative group showed lower average scores of all four kinds of stigma compared to those with other non-normative identities, with internalized, experienced and anticipated stigma in relationships scores contributing most strongly.

A possible explanation for these results might be that in many Western societies, the acceptance of different sexual orientations has increased in recent decades, due in part to legal recognition, decriminalization and visible emancipation movements (Wike et al., 2013). Positive media attention and representation in films, television and politics have also contributed to further normalization. This exposure and personal contact with gay, lesbian and bisexual people generally leads to more positive attitudes (Pettigrew and Tropp, 2006). Belgium, in which this study was conducted, is specifically known for its LGBTQIA+ friendly legislation (Eeckhout and Paternotte, 2011).

In contrast, TGNC people’s identity has historically been more medicalized and pathologized (Reisner et al., 2016), and attitudes among heterosexual individuals remain more negative towards TGNC people compared to LGB people (Norton and Herek, 2013). In another comparison, a recent study by Hansen-Brown and Jefferson (2022) found that the general population stigmatized BDSM practitioners more than the gay/lesbian population, although both were stigmatized more than a low-stigma comparison group (i.e., people in a romantic relationship).

This also shows that stigma towards LGB individuals may not be discounted. Negative attitudes are still found in Western countries such as Belgium, specifically associated with religiosity and more likely to come from men (Hooghe et al., 2010; Norton and Herek, 2013). It must also be acknowledged that the anti-gender mobilization currently observed in Belgium and other Western countries also poses a threat for the LGBTQIA+ community as a whole (Verlooy, 2024).

### Stigmatization of non-normative sexual relationships

This study found that people engaged in non-normative sexual relationships comprised about 6.7% of all included participants. The group was generally younger in age and more likely to be male or non-binary compared to those with a normative sexual relationship. Further analyses conducted within the non-normative group showed lower average scores of experienced stigma and anticipated stigma in daily life and higher average scores for anticipated stigma in relationships and internalized stigma compared to those with other non-normative identities. Experienced stigma and anticipated stigma in daily life scores contributing most strongly, although these were still found to be weak correlations.

These results may reflect the societal taboo surrounding alternative sexual practices such as BDSM or open relationships which fosters a fear of discrimination in their social relationships and has an impact on how they view themselves. However, because having a non-normative sexual relationship can be considered a CSI, it stands to reason that experienced stigma would be lower in this group. This may also explain why they experience higher rates of anticipated stigma in their relationships compared to their daily life, seeing as this identity will more at risk to be revealed within intimate relationships.

As mentioned above, Hansen-Brown and Jefferson (2022) found indications that the BDSM population is more stigmatized than the gay/lesbian population, especially in sectors like healthcare. This is corroborated by a study from Schuerwegen et al. (2020), which found that about 86% of the general population agreed with at least one stigmatizing attitude towards BDSM practitioners. The same study found that about 28% of the BDSM-participants reported not feeling comfortable to share their interests with the outside world (Schuerwegen et al., 2020). Because of this, they will generally try to conceal their identity. Quinn and Chaudoir (2009) have shown that the possibility of hiding a stigmatized identity is not necessarily less stressful than having a visible identity. The option to hide something leads to additional cognitive load and social uncertainty, which can make the level of anticipated stigma relatively high compared to more visible forms of stigma.

### Stigmatization of non-normative romantic relationships

This study found that people engaged in non-normative romantic relationships comprised about 3.6% of all included participants. The group was generally younger in age, more likely to be male or non-binary and to have a higher educational level compared to those with a normative romantic relationship. Further analyses within the non-normative group showed slightly higher average scores of all four kinds of stigma compared to those with other non-normative identities, although these scores were found to be not significantly differing.

Because of the more public nature of romantic relationships compared to sexual relationships, it may be assumed that people with non-normative romantic relationships are less concealed than those with non-normative sexual relationships, which might explain why they—in accordance with TGNC people—experience higher levels of discrimination and internalized stigma (Moors et al., 2021; Rodrigues et al., 2024).

For example, Young (2014) found that non-monogamist people managed their stigmatized identities differently depending upon whether they were doing so in their private or public life. Those respondents described how they constantly anticipated negative reactions and developed public management strategies to avoid or reduce stigma. Some participants showed signs of internalized stigma through doubts about their own worthiness or the feeling of being ‘different’ or ‘less legitimate’.

Research by Mahar et al. (2024) had similar findings: of the participants who reported that they did not experience stigma, approximately 40% reported that they limited disclosure of their CNM, often to avoid being treated differently. This aligns with a recent qualitative study which found that many CNM people perceived that others viewed them as deviant, which led to limited or nondisclosure of their CNM identity to avoid stigma (O’Byrne and Haines, 2021). Their second study showed that experienced stigma is positively associated with psychological distress, with anticipated and internalized stigma partially mediating this relationship. Experiencing stigma could lead to anticipating more stigma and internalizing this stigma, which could then lead to lower psychological wellbeing. This suggests that the anticipation of negative reactions and the internalization of negative beliefs about polyamory contribute to increased psychological distress.

### Stigmatization of multiple non-normative identities

This study found that about 80% of the non-normative group had a single non-normative identity, while about 20% had two or more non-normative identities. Further analyses within this non-normative group showed that while the first group had higher average scores of experienced and internalized stigma, the second group had higher average scores of anticipated stigma in daily life and relationships, with internalized and experienced stigma scores contributing most strongly.

These results suggest that stigma is mediated differently when a person belongs to more than one minority community. It may be hypothesized that having multiple non-normative identities leads to a more integrated community where exposure to discrimination is less. For instance, Sprott (2023) discussed how involvement in BDSM practices could have positive outcomes for LGBTQ+ individuals, including reducing internalized stigma. The author noted that LGBTQ+ individuals were more likely to participate in kink activities and that this involvement could contribute to personal growth, self-acceptance, and overcoming negative feelings that stem from societal stigma. By actively participating in kink communities, these individuals may experience a sense of autonomy and empowerment, which helps to combat internalized stigma.

Furthermore, a slight correlation was found between multiple non-normative identities and higher rates of anticipated stigma. Reinka et al. (2020) found something similar when looking specifically at CSI’s. When multiple CSI’s were present, their participants reported higher rates of anticipated stigma, which may be attributed to the struggle of having to hide not one but several parts of one’s identity. This also predicted a worse outcome of health, in line with the minority stress model of Meyer (2003).

### Clinical implications

Although recent years have seen an increase in societal awareness of sexual and gender minority groups and the stress to which they are subjected, stigmatization is not a problem of the past. In the current political landscape, with many countries voting for policies which further limit the rights of sexual and gender minorities, recent statistics are already showing an increase in hate crimes and violence towards LGBTQIA+ people (Frost and Meyer, 2023). The authors further cite an increase in reported minority stress among young people, with important societal implications.

Stigma in non-normative groups therefore remains an important social theme for policymakers. This can be done, for example, by drafting legislation that explicitly addresses the rights of people with these identities and by creating more societal awareness through media and education. The education of healthcare workers is equally important in further combatting stigmatizing attitudes. Research has been done in that regard, for instance to summarize existing LGBTQIA+ cultural competency in healthcare (Kuney et al., 2025) or offer clinical considerations in treating people who practice BDSM (Dunkley and Brotto, 2018).

To further these aims, more intersectional research needs to be done, focusing on how different stigmatizing factors accumulate across diverse non-normative groups. While many existing studies focus on one specific minority group, this research highlights the need to examine combinations and the interactions between different types of stigma and different non-normative identities. Part of future research should consider the role of social networks and support structures in reducing stigma, as these can promote people’s mental health and overall well-being. Qualitative research will also be invaluable to better explore the fluidity of identity as it relates to intersectionality, seeing as non-normative identities will express differently depending on contextual and personal factors and will furthermore change throughout the lifetime of the individual.

### Limitations

One of the core strengths of this study also underlines a limitation. Because multiple groups are included here, both overlapping and not overlapping, some subgroups were relatively small with a possible impact on the statistical power. Larger populations in future research might address this limitation. This may also give an opportunity to refine the included non-normative identities to further investigate the intersectionality. It must be acknowledged that this study design does not capture an in depth exploration of intersectionality because it does not allow for the fluidity of identity, both in time and depending on the context. Other studies will be necessary to build upon this research.

Finally, the study was only conducted in Dutch and distributed among a Flemish population. This means that the results are culturally specific and only include a WEIRD (Western, Educated, Industrialized, Rich, Democratic) population (Gurven and Lieberman, 2020).

## Conclusion

By including a large cross-section of the Flemish population, this research was able to study four minority groups which have been previously linked to each other, but which also present with different identities and challenges within society. Subtle differences in the kinds of stigma these groups face were found. The more visible minority groups such as those with a non-normative gender identity or romantic relationship faced more stigma on all fronts. The generally more accepted minority of people with a non-normative sexual orientation generally faced less stigma than other minority groups included. Those with the more concealable minority such as people with non-normative sexual orientations experienced less stigma, though they did suffer from higher degrees of anticipated and internalized stigma. In general, having less non-normative identities was associated with less anticipated stigma, though they were more likely to suffer from discrimination and internalized stigma. These results shed light on the importance of intersectionality and the impact we have as a society.

## Data Availability

The raw data supporting the conclusions of this article will be made available by the authors, without undue reservation.

## References

[ref1] Barton M. Yeatts P. E. Henson R. K. Martin S. B. (2016). Moving beyond univariate post-hoc testing in exercise science: a primer on descriptive discriminate analysis. Res. Q. Exerc. Sport 87, 365–375. doi: 10.1080/02701367.2016.1213352, 27548736

[ref2] Bos A. E. Pryor J. B. Reeder G. D. Stutterheim S. E. (2013). Stigma: advances in theory and research. Basic Appl. Soc. Psychol. 35, 1–9. doi: 10.1080/01973533.2012.746147

[ref3] Brown A. Barker E. D. Rahman Q. (2020). A systematic scoping review of the prevalence, etiological, psychological, and interpersonal factors associated with BDSM. J. Sex Res. 57, 781–811. doi: 10.1080/00224499.2019.1665619, 31617765

[ref4] Buchanan N. T. Wiklund L. O. (2021). Intersectionality research in psychological science: resisting the tendency to disconnect, dilute, and depoliticize. Res. Child Adolesc. Psychopathol. 49, 25–31. doi: 10.1007/s10802-020-00748-y, 33400076

[ref5] Clark K. D. Jewell J. Sherman A. D. F. Balthazar M. S. Murray S. B. Bosse J. D. (2025). Lesbian, gay, bisexual, transgender and queer people's experiences of stigma across the spectrum of inpatient psychiatric care: a systematic review. Int. J. Ment. Health Nurs. 34:e13455. doi: 10.1111/inm.13455, 39435958 PMC11771681

[ref6] De Neef N. Coppens V. Huys W. Morrens M. (2019). Bondage-discipline, dominance-submission and sadomasochism (BDSM) from an integrative biopsychosocial perspective: a systematic review. Sex Med. 7, 129–144. doi: 10.1016/j.esxm.2019.02.002, 30956128 PMC6525106

[ref7] Doyle D. M. Begeny C. T. Barreto M. Morton T. A. (2021). Identity-related factors protect well-being against stigma for transgender and gender non-conforming people. Arch. Sex. Behav. 50, 3191–3200. doi: 10.1007/s10508-021-02029-1, 34613539 PMC8563541

[ref8] Drabish K. Theeke L. A. (2022). Health impact of stigma, discrimination, prejudice, and Bias experienced by transgender people: a systematic review of quantitative studies. Issues Ment. Health Nurs. 43, 111–118. doi: 10.1080/01612840.2021.1961330, 34469283

[ref9] Dunkley C. R. Brotto L. A. (2018). Clinical considerations in treating BDSM practitioners: a review. J. Sex Marital Ther. 44, 701–712. doi: 10.1080/0092623X.2018.1451792 29543573

[ref10] Eeckhout B. Paternotte D. (2011). A paradise for LGBT rights? The paradox of Belgium. J. Homosex. 58, 1058–1084. doi: 10.1080/00918369.2011.598414, 21902492

[ref11] Flentje A. Heck N. C. Brennan J. M. Meyer I. H. (2020). The relationship between minority stress and biological outcomes: a systematic review. J. Behav. Med. 43, 673–694. doi: 10.1007/s10865-019-00120-6, 31863268 PMC7430236

[ref12] Frost D. M. Meyer I. H. (2023). Minority stress theory: application, critique, and continued relevance. Curr. Opin. Psychol. 51:101579. doi: 10.1016/j.copsyc.2023.101579, 37270877 PMC10712335

[ref13] Furuya K. (2002). A socio-economic model of stigma and related social problems. J. Econ. Behav. Organ. 48, 281–290. doi: 10.1016/S0167-2681(01)00231-1

[ref14] Gamariel F. Isaakidis P. Tarquino I. A. P. Beirao J. C. O'Connell L. Mulieca N. . (2020). Access to health services for men who have sex with men and transgender women in Beira, Mozambique: a qualitative study. PLoS One 15:e0228307. doi: 10.1371/journal.pone.0228307, 31999760 PMC6992190

[ref15] Goffman E. (1963). Stigma: notes on the management of spoiled identity. London, United Kingdom: Penguin Books.

[ref16] Gurven M. D. Lieberman D. E. (2020). WEIRD bodies: mismatch, medicine and missing diversity. Evol. Hum. Behav. 41, 330–340. doi: 10.1016/j.evolhumbehav.2020.04.001, 33100820 PMC7584376

[ref17] Hansen-Brown A. A. Jefferson S. E. (2022). Perceptions of and stigma toward BDSM practitioners. Curr. Psychol. 42, 1–9. doi: 10.1007/s12144-022-03112-z, 35496362 PMC9041285

[ref18] Harari L. Lee C. (2021). Intersectionality in quantitative health disparities research: a systematic review of challenges and limitations in empirical studies. Soc. Sci. Med. 277:113876. doi: 10.1016/j.socscimed.2021.113876, 33866085 PMC8119321

[ref19] Henderson E. R. Goldbach J. T. Blosnich J. R. (2022). Social determinants of sexual and gender minority mental health. Curr. Treat. Options Psych. 9, 229–245. doi: 10.1007/s40501-022-00269-z

[ref20] Hooghe M. Claes E. Harell A. Quintelier E. Dejaeghere Y. (2010). Anti-gay sentiment among adolescents in Belgium and Canada: a comparative investigation into the role of gender and religion. J. Homosex. 57, 384–400. doi: 10.1080/00918360903543071, 20391000

[ref21] James S. E. Herman J. L. Rankin S. Keisling M. Mottet L. Anafi M. (2016). The report of the 2015 U.S. transgender survey. Available online at: http://www.ustranssurvey.org/reports

[ref22] Jefferson K. Neilands T. B. Sevelius J. (2013). Transgender women of color: discrimination and depression symptoms. Ethn. Inequal Health Soc. Care 6, 121–136. doi: 10.1108/EIHSC-08-2013-0013, 25346778 PMC4205968

[ref23] Kessler R. C. Mickelson K. D. Williams D. R. (1999). The prevalence, distribution, and mental health correlates of perceived discrimination in the United States. J. Health Soc. Behav. 40, 208–230. doi: 10.2307/2676349 10513145

[ref24] Kuney M. A. Noble M. D. Stubbs D. M. (2025). LGBTQIA+ cultural competency in healthcare education programs: a scoping review. Nurse Educ. Pract. 84:104333. doi: 10.1016/j.nepr.2025.104333, 40174474

[ref25] Lecuona O. Suero M. Wingen T. de Rivas S. (2021). Does “open” rhyme with “special”? Comparing personality, sexual satisfaction, dominance and jealousy of monogamous and non-monogamous practitioners. Arch. Sex. Behav. 50, 1537–1549. doi: 10.1007/s10508-020-01865-x, 33942196

[ref26] Liss M. Derflinger T. Wilson L. (2024). Student resources and retention among transgender and nonbinary college students. Divers. Incl. Res. 1:e70002. doi: 10.1002/dvr2.70002

[ref27] Livingston J. D. Boyd J. E. (2010). Correlates and consequences of internalized stigma for people living with mental illness: a systematic review and meta-analysis. Soc. Sci. Med. 71, 2150–2161. doi: 10.1016/j.socscimed.2010.09.030, 21051128

[ref28] Mahar E. A. Irving L. H. Derovanesian A. Masterson A. Webster G. D. (2024). Stigma toward consensual non-monogamy: thematic analysis and minority stress. Personal. Soc. Psychol. Bull. 50, 571–586. doi: 10.1177/01461672221139086, 36461779

[ref29] Meyer I. H. (2003). Prejudice, social stress, and mental health in lesbian, gay, and bisexual populations: conceptual issues and research evidence. Psychol. Bull. 129, 674–697. doi: 10.1037/0033-2909.129.5.674, 12956539 PMC2072932

[ref30] Mezza F. Mezzalira S. Pizzo R. Maldonato N. M. Bochicchio V. Scandurra C. (2024). Minority stress and mental health in European transgender and gender diverse people: a systematic review of quantitative studies. Clin. Psychol. Rev. 107:102358. doi: 10.1016/j.cpr.2023.102358, 37995435

[ref31] Michaels E. Thomas M. Reeves A. Price M. Hasson R. Chae D. . (2019). Coding the everyday discrimination scale: implications for exposure assessment and associations with hypertension and depression among a cross section of mid-life African American women. J. Epidemiol. Community Health 73, 577–584. doi: 10.1136/jech-2018-211230, 30894420 PMC7200149

[ref32] Miller L. R. Grollman E. A. (2015). The social costs of gender nonconformity for transgender adults: implications for discrimination and health. Sociol Forum (Randolph N J) 30, 809–831. doi: 10.1111/socf.12193, 27708501 PMC5044929

[ref33] Moors A. C. Schechinger H. A. Balzarini R. Flicker S. (2021). Internalized consensual non-monogamy negativity and relationship quality among people engaged in polyamory, swinging, and open relationships. Arch. Sex. Behav. 50, 1389–1400. doi: 10.1007/s10508-020-01885-7, 34100145

[ref34] Newcomb M. E. Hill R. Buehler K. Ryan D. T. Whitton S. W. Mustanski B. (2020). High burden of mental health problems, substance use, violence, and related psychosocial factors in transgender, non-binary, and gender diverse youth and young adults. Arch. Sex. Behav. 49, 645–659. doi: 10.1007/s10508-019-01533-9, 31485801 PMC7018588

[ref35] Norton A. T. Herek G. M. (2013). Heterosexuals’ attitudes toward transgender people: findings from a National Probability Sample of U.S. adults. Sex Roles 68, 738–753. doi: 10.1007/s11199-011-0110-6

[ref36] O’Byrne P. Haines M. (2021). A qualitative exploratory study of consensual non-monogamy: sexual scripts, stratifications and charmed circles. Soc. Theory Health 19, 137–154. doi: 10.1057/s41285-019-00120-1

[ref37] Pettigrew T. F. Tropp L. R. (2006). A meta-analytic test of intergroup contact theory. J. Pers. Soc. Psychol. 90, 751–783. doi: 10.1037/0022-3514.90.5.751, 16737372

[ref38] Quinn D. M. Chaudoir S. R. (2009). Living with a concealable stigmatized identity: the impact of anticipated stigma, centrality, salience, and cultural stigma on psychological distress and health. J. Pers. Soc. Psychol. 97, 634–651. doi: 10.1037/a0015815, 19785483 PMC4511710

[ref39] Quinn D. M. Williams M. K. Weisz B. M. (2015). From discrimination to internalized mental illness stigma: the mediating roles of anticipated discrimination and anticipated stigma. Psychiatr. Rehabil. J. 38, 103–108. doi: 10.1037/prj0000136, 25844910 PMC4469573

[ref40] Reinka M. A. Pan-Weisz B. Lawner E. K. Quinn D. M. (2020). Cumulative consequences of stigma: possessing multiple concealable stigmatized identities is associated with worse quality of life. J. Appl. Soc. Psychol. 50, 253–261. doi: 10.1111/jasp.12656

[ref41] Reisner S. L. Poteat T. Keatley J. Cabral M. Mothopeng T. Dunham E. . (2016). Global health burden and needs of transgender populations: a review. Lancet 388, 412–436. doi: 10.1016/S0140-6736(16)00684-X, 27323919 PMC7035595

[ref42] Rodrigues D. L. Brooks T. R. Balzarini R. N. Moors A. C. Lopes D. (2024). Examining the role of mononormative beliefs, stigma, and internalized consensual non-monogamy negativity for dehumanization. Arch. Sex. Behav. 53, 889–899. doi: 10.1007/s10508-023-02785-2, 38182813

[ref43] Rubel A. N. Burleigh T. J. (2020). Counting polyamorists who count: prevalence and definitions of an under-researched form of consensual nonmonogamy. Sexualities 23, 3–27. doi: 10.1177/1363460718779781

[ref44] Schuerwegen A. De Zeeuw I. Huys W. Henckens J. Goethals K. Morrens M. (2020). A survey study investigating stigma towards BDSM in the general population and self-stigmatization among BDSM practitioner. Sex. Med. 4. doi: 10.47739/2578-3718/1055

[ref45] Sprott R. A. (2023). The intersection of LGBTQ+ and kink sexualities: a review of the literature with a focus on empowering/positive aspects of kink involvement for LGBTQ+ individuals. Curr. Sex. Health Rep. 15, 107–112. doi: 10.1007/s11930-023-00360-3

[ref46] Tebbe E. A. Simone M. Wilson E. Hunsicker M. (2022). A dangerous visibility: moderating effects of antitrans legislative efforts on trans and gender-diverse mental health. Psychol. Sex. Orientat. Gend. Divers. 9, 259–271. doi: 10.1037/sgd0000481, 36188191 PMC9518927

[ref47] Traeen B. Thuen F. (2022). Non-consensual and consensual non-monogamy in Norway. Int. J. Sex. Health 34, 65–80. doi: 10.1080/19317611.2021.1947931, 38595687 PMC10906970

[ref48] Valentine S. E. Shipherd J. C. (2018). A systematic review of social stress and mental health among transgender and gender non-conforming people in the United States. Clin. Psychol. Rev. 66, 24–38. doi: 10.1016/j.cpr.2018.03.003, 29627104 PMC6663089

[ref49] Velasco R. A. F. (2022). Stigma among transgender and gender-diverse people accessing healthcare: a concept analysis. J. Adv. Nurs. 78, 698–708. doi: 10.1111/jan.15040, 34524708

[ref50] Ventriglio A. Bhugra D. (2019). Sexuality in the 21st century: sexual fluidity. East Asian Arch. Psychiatr. 29, 30–34. doi: 10.12809/eaap1736, 31237255

[ref51] Verlooy R. (2024). Anti-gender mobilizations and transgender rights: unpacking recent evolutions in Belgium. Tijdschr. Genderstud. 27, 351–374. doi: 10.5117/TVGN2024.4.004.VERL

[ref52] Wike R. Horowitz J. M. Simmons K. Poushter J. Ponce A. Barker C. . (2013). The global divide on homosexuality: greater acceptance in more secular and affluent countries. Washington DC, United States.

[ref53] Williams D. R. Yu Y. Jackson J. S. Anderson N. B. (1997). Racial differences in physical and mental health: socioeconomic status, stress, and discrimination. J. Health Psychol. 2, 335–351. doi: 10.1177/135910539700200305, 22013026

[ref54] Winter S. Diamond M. Green J. Karasic D. Reed T. Whittle S. . (2016). Transgender people: health at the margins of society. Lancet 388, 390–400. doi: 10.1016/S0140-6736(16)00683-8, 27323925

[ref55] Young J. M. (2014). “We are pioneers”: Polyamorists’ stigma management strategies (publication number paper 533) University of Missouri-Saint Louis. Available online at: http://opensiuc.lib.siu.edu/gs_rp/533

